# Parkin increases the risk of colitis by downregulation of VDR via autophagy-lysosome degradation

**DOI:** 10.7150/ijbs.77153

**Published:** 2023-03-05

**Authors:** Zemeng Ma, Junxian Wu, Yuqing Wu, Xiaomeng Sun, Zebing Rao, Naishuang Sun, Yu Fu, Zaikui Zhang, Jingzhou Li, Mengjun Xiao, Qiang Zeng, Yuxuan Wu, Chenhua Han, Ding Ding, Hongjie Zhang, Hua Yuan, Jing zhang, Shuo Yang, Yunzi Chen

**Affiliations:** 1 Key Laboratory of Immune Microenvironment and Disease, Department of Immunology, Nanjing Medical University, Nanjing211166, China.; 2 Medical Centre for Digestive Diseases, Second Affiliated Hospital of Nanjing Medical University, Nanjing 211166, China.; 3 The State Key Laboratory of Pharmaceutical Biotechnology, School of Life Sciences, Nanjing University, Nanjing 210023, China.; 4 Department of Gastroenterology, The First Affiliated Hospital of Nanjing Medical University, Nanjing 211166, China.; 5 Department of Laboratory Medicine, The First Affiliated Hospital of Nanjing Medical University, Nanjing211166, China.; 6 National Key Clinical Department of Laboratory Medicine, Nanjing 210029, China.; 7 Jiangsu Key Laboratory of Oral Diseases, Nanjing Medical University, Nanjing, China.; 8 Department of Oral and Maxillofacial Surgery, Affiliated Hospital of Stomatology, Nanjing Medical University, Nanjing, China.

**Keywords:** Parkin, Vitamin D receptors, inflammatory bowel diseases

## Abstract

Parkin, an E3 ubiquitin ligase, plays an essential role in mitophagy. Emerging evidence indicates that mitophagy is involved in various processes closely related to immune diseases, including inflammatory bowel diseases (IBD). Here, the authors show that Parkin increases the occurrence of colitis and severe inflammation. Deletion of Parkin resulted in marked reductions in colonic inflammation and exhibited high resistance to DSS-induced colitis. Mechanism investigation indicated that Parkin interacts with Vitamin D receptors (VDR), a critical inhibitory regulator in IBD. Parkin promotes VDR degradation via the p62-related autophagy-lysosome pathway. Comparison of colitis in Parkin-/- and Parkin-/-Vdr-/- mice showed that the protective effect of Parkin deletion against colitis was abolished by VDR deletion. The result suggests that the regulatory effect of Parkin in colitis is a VDR-dependent pathway. Our research provides a new role of Parkin in colitis by downregulating VDR, which provides a potential strategy for treating IBD.

## Introduction

Inflammatory bowel disease (IBD), characterized by gastrointestinal inflammation, is a group of autoimmune diseases. The two main clinical manifestations are Crohn's disease (CD) and ulcerative colitis (UC). The immune pathogenesis of IBD involves the immune system's interaction with environmental, genetic, vitamin D, and microbial factors 1-4. Mitochondrial stress-induced mitophagy plays an anti-inflammatory role in IBD 5,6; Paneth cell abnormalities have been associated with defective mitophagy and autophagy in murine intestinal cells 7; Mitophagy also influences intestinal epithelial cell (IEC) viability 8. Impaired mitochondrial integrity stimulates mitophagy via enhanced intracellular ROS in IECs of IBD patients 9. Thus, mitophagy is a protective process in IBD by regulating pro-inflammatory cytokine production, pathogen clearance, and IEC viability.

Mitochondrial damage is involved in Parkinson's disease (PD). Parkin (PARK2) mutations are identified in patients with PD and increase the susceptibility to Parkinson's disease 10. Parkin is an E3 ubiquitin ligase with an amino-terminal ubiquitin-like (Ubl) domain and a carboxyl-terminal ubiquitin ligase domain 11. PINK1 accumulates on the outer membrane of damaged mitochondria, activates Parkin's E3 ubiquitin ligase activity, and recruits Parkin to the dysfunctional mitochondrion 12. When mitochondria are damaged or depolarized, Pink1 phosphorylates the Parkin Ubl domain Ser65 to activate Parkin, and Parkin modifies many cytosolic and OMM (mitochondrial outer membrane) proteins with K48- and K63-linked ubiquitin chains. Ubiquitinated OMM proteins containing K63-linked polyubiquitin chains are recognized by the specific autophagy receptor P62, which recruits LC3 and triggers mitophagy 12. Pink1-Parkin regulates mitochondrial homeostasis, anti-oxidative stress, and mitophagy 13. At ten months of age, Parkin KO mice displayed multiple signs of inflammation, and Parkin deficiency enhanced inflammation via potentiating the RIPK1-RIPK3 interaction in necroptosis 14. The role of Parkin in the pathogenesis of IBD is poorly understood.

To reveal the possible role of Parkin contributing to IBD, we screened the proteins associated with Parkin in the epithelial cells of the intestine. In this study, we report that VDR (Vitamin D receptor) is a novel Parkin-associated protein. VDR is highly expressed in intestinal epithelial cells (IECs) and regulates the biological activity of vitamin D (1,25-dihydroxy vitamin D) 15. Gut epithelial VDR signaling inhibits colitis by protecting the mucosal epithelial barrier 16. Clinical evidence has shown that Vitamin D receptors (VDR) signals are closely related to IBD development 17. VDR deficiency in the intestine increases the susceptibility to experimental colitis 18. Our results indicated that Parkin promotes VDR degradation via the autophagy-lysosome pathway. The Parkin-VDR axis plays a critical regulation in the IECs involved in the pathogenesis of IBD.

## Materials and Methods

### Mice

Vdr-/- and Parkin-/- mice have been described previously 19,20. Vdr-/- mice were crossed with Parkin-/-mice to generate Vdr-/- Parkin-/- homozygous knockouts. The Institutional Animal Care and Use Committee of Nanjing Medical University approved all animal handling and procedures performed in this study. Mice were used at 8-12 weeks of age. All efforts were made to minimize animal suffering and reduce the number of animals used.

### Antibodies and reagents

Anti-VDR (sc-13133), Anti-Parkin (sc-32282), and Anti-β-Actin (sc-8432) were brought from Sigma-Aldrich. Anti-HA-Tag (#2367) and Anti-LC3 (#4108) were from Cell Signaling Technology. Anti-MYC-tag (10828-1-AP), Anti-GAPDH (60004-1-Ig), and Anti-P62 (18420-1-AP) were from Proteintech. Anti-FLAG was brought from Sigma(F7425). Anti-laminB (AF1408) was brought from Beyotime Biotechnology. DSS (DB001-38) was from TdB Consultancy. Anti-Cy3-AffiniPureGoat Anti-Mouse IgG (H+L), Anti-Cy3-AffiniPure Goat Anti-Rabbit IgG (H+L), Anti- FITC-AffiniPure Goat Anti-Mouse IgG (H+L), and Anti- FITC-AffiniPure Goat Anti-Rabbit IgG (H+L) were from Jackson ImmunoResearch.

### Bone marrow chimeras

C57BL/6 WT mice or Parkin-/-mice at six weeks were reconstituted with 5× 10^ 6^ bone marrow cells from WT mice or Parkin-/-mice. Recipient mice received two 5.5Gy irradiation three h apart and were treated with Neomycin for three weeks. After irradiation12 weeks, mice were treated with DSS.

### Parkin-associated proteins Mass Spectrometry analysis

HCT116 cells were transfected with empty Flag vector or Flag-Parkin for 24 hrs, then collected and resuspended in lysis buffer (2% (w/v) SDS,50 mM Tris-HCl (pH 6.8), 100 mM DTT (dithiothreitol), 0.1% (w/v) bromophenol blue and 10% (v/v) glycerol). Extracts were immunoprecipitated with anti-Flag antibody and Protein A/G-Agarose beads, and the IP pulldown protein complex was separated with SDS-PAGE, visualized by coomassie blue staining, then excised in-gel digestion with trypsin. The peptides extracted from gel bands were subject to LC-MS/MS analysis. The original MS/MS data were analyzed with Protein Pilot Software (version 4.5 AB Sciex) and searched in the UniProt database against Homo sapiens.

### Immunofluorescence Staining

For fluorescence colocalization analysis, Hela was plated on coverslips. The cells were fixed for 20min with 4% paraformaldehyde and then permeabilized with 0.2% NP-40/PBS for 10min. The cells were incubated overnight, followed by anti-rabbit Cy3-conjugated AffiniPure (Jackson ImmunoResearch). DAPI (4′,6′-diamidino-2-phenylindole hydrochloride; Sigma-Aldrich) stained Nuclei. HEK293T cells transiently transfected with plasmids encoding, cultured for 24 h. The cells were incubated overnight with antibodies and then with fluorescent secondary antibodies using confocal laser microscopy (LSM710, Carl Zeiss, Germany) to examine.

### Immunoprecipitation and Immunoblot

For whole-cell lysate analysis, the cell lysis buffer is SDS [50 mM Tris-HCl (pH 6.8), 0.1% (w/v) blue bromophenol, 10% (w/v) Glycerol, 2% (w/v) SDS, and 100 mM DTT]. For immunoprecipitation, treat the cells as directed and place the cells in 500 µl RIPA [50 mM Tris (pH 7.4) lysis buffer, 150 mM NaCl, 1% NP-40, and 0.25% sodium deoxycholate]. Collect and collect within 30 minutes at 4°C. Centrifuge at 12,000 g for 10 minutes, then collect the supernatant and incubate with the appropriate antibody overnight. Add 10 µl of Protein A/G Magnetic Beads (MCE HY-K0202) and spin gently for 90 minutes at four °C to capture immune complexes.

Furthermore, spin gently for 90 minutes at 4 °C to capture immune complexes, then centrifuge at 1000 rpm for 3 minutes to collect the agarose beads. The supernatant was discarded, and then washing the beads were three times with 1000 µl ice-cold RIPA buffer and two times with PBS. Then, resuspend the agarose beads in 50 µl SDS Loading Buffer and mix gently. Separating sample with SDS-PAGE, transferred sample to a polyvinylidene fluoride (PVDF) membrane (Millipore), and analyzed by immunoblotting. The Tanon imaging system visualizes the immune response.

### DSS-induced colitis

WT and Parkin-/-mice were treated with 2.5% DSS for seven days to induce acute experimental colitis, then drank water for two days. During the experiment, monitor the body weight, stool, and body posture daily to assess the disease activity index (DAI). DAI is a total score of weight loss and starting weight, stool stability, and body posture. The scores are as follows: weight loss: 0 (no loss), 1 (5 to 10%), 2 (10 to 15%), and 3 (> 15%); stool consistency: 0 (standard), 1 (soft stool), 2 (Soft stool and blood in the stool) and 3 (diarrhea and blood in the stool); body posture: 0 (thicken without smooth hair), 1 (light hair and crutches), 2 (medium hair and crutches), and 3 (heavy hair and heavy flushing). The mice were euthanized on the ninth day and immediately collected for colon length measurement, cytokines analysis, and histological analysis.

### Real-time quantitative polymerase chain reaction

Total RNA was extracted using TRIzol (Life) reagent, and complementary DNA synthesis was performed. The reverse transcript of the various samples was used by StepOnePlus (Applied Biosystems) under the direction of the Brilliant SYBR Green QPCR Master Mix (Vazyme) manufacturer. The primers used are listed in Table 1.

### shRNA-mediated gene silencing

shParkin5'GGATCAGCAGAGCATTGTTCA 3' and ShP62 5'GCAGATGAGAAAGATCGCCTT 3' were generated into vector pRNA-U6.1, then transfected them into HCT116 cells with PolyJet^TM^ (SignaGen) in OPTI-MEM. The medium was changed 6 h later, and the cells were cultured for 48 h in DMEM medium supplemented with 10% FBS.

### Histological analysis and immunohistochemical staining

Freshly dissected colon or colon biopsies were fixed overnight with 4% formaldehyde in PBS (pH 7.2), processed, and embedded in paraffin wax. Tissues were cut into 4-μm sections. Colonic morphology was examined by H&E staining. To examine VDR localization in the colon, we stained the slices with anti-VDR (Santa Cruz Biotechnology Inc.) as primary antibodies, followed by staining with horseradish peroxidase-conjugated anti-IgG as second antibodies. Antigen was then visualized with the 3,3′-diaminobenzidine substrate (Sigma-Aldrich) and observed under a light microscope.

### Cytoplasmic protein and nuclear protein extraction

The nuclear protein and Cytosol protein were prepared using the nuclear/Cytosol protein extraction kit (BeyotimeP0027).

### Statistical analyses

The data were analyzed by GraphPad Prism 8.0 software and expressed as mean±SEM. Using two-sided unpaired t-tests or multiple-group two-way analysis of variance (ANOVA) to analyze statistical data. *P*-value is expressed as *P < 0.05; **P < 0.01; *** *P* < 0.001.

## Results

### Parkin-/- mouse attenuates DSS-induced colitis

**Figure 1:**
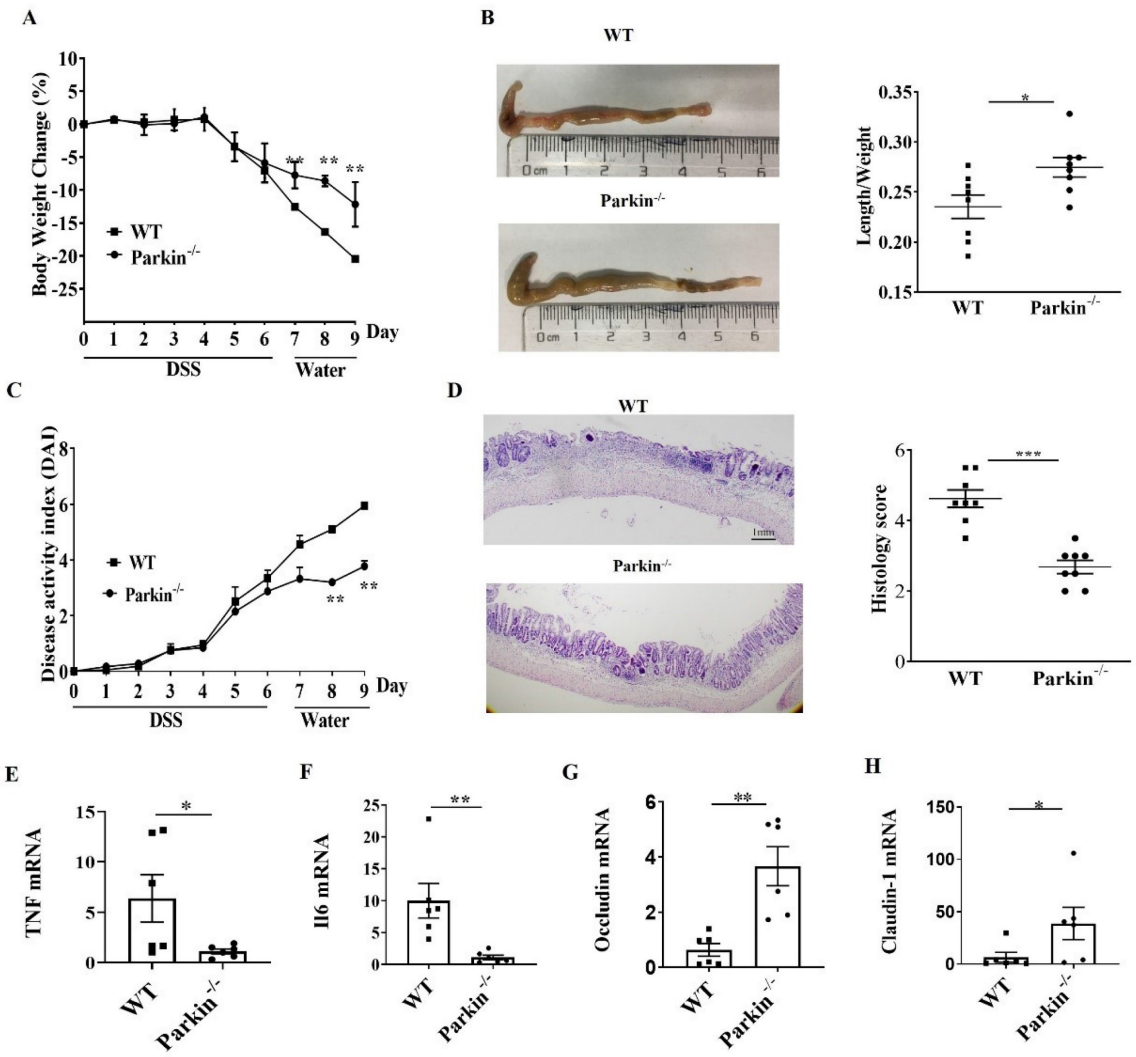
** Parkin-/- mouse attenuates DSS induced colitis.** WT and Parkin-/-mice were given 2.5% (w/v) DSS for six days, followed by normal drinking water for three days, and disease severity was measured according to a percentage weight loss(A), colon length(B), Disease activity index (DAI)(C), H&E-stained sections of the colon(D) and histology score (E), scale bar=1mm. Quantitating pro-inflammatory cytokines TNFɑ(F), IL-6(G), and tight junction protein Occludin(H), Claudin-1(I) in colonic mucosa from control and DSS-treated WT and Parkin-/-mice on day 9 with Real-time RT-PCR. Results are presented as means±SEM.*P < 0.05, **P < 0.01, *** *P* < 0.001 versus the corresponding WT. n = 6-7 in each genotype.

To examine the role of Parkin in IBD, we assessed the occurrence of IBD in the experimental model of DSS-induced colitis in WT and Parkin-/- mice. The loss of body weight and shortening of the colon were consistently alleviated in Parkin-/- mice compared with WT mice (Fig. 1A and 1B). The disease activity index (DAI) and histological examination also showed the relief of colitis in Parkin-/- mice (Fig. 1C and 1D). Similarly, colonic pro-inflammatory cytokines (TNFa and IL-6) in Parkin-/- mice were lower in WT mice with DSS-induced colitis (Fig.1E and 1F). Furthermore, we detected the tight junction-related proteins Occludin and Claudin-1, which are essential for the integrity of the intestinal barrier. The result showed that Occludin and Claudin-1 mRNA were significantly upregulated in Parkin-/- mice (Fig. 1G, 1H), suggesting that loss of Parkin has a protective effect in DSS-induced colitis.

### Parkin-/- reduces DSS-induced colitis independent of immune cells

**Figure 2:**
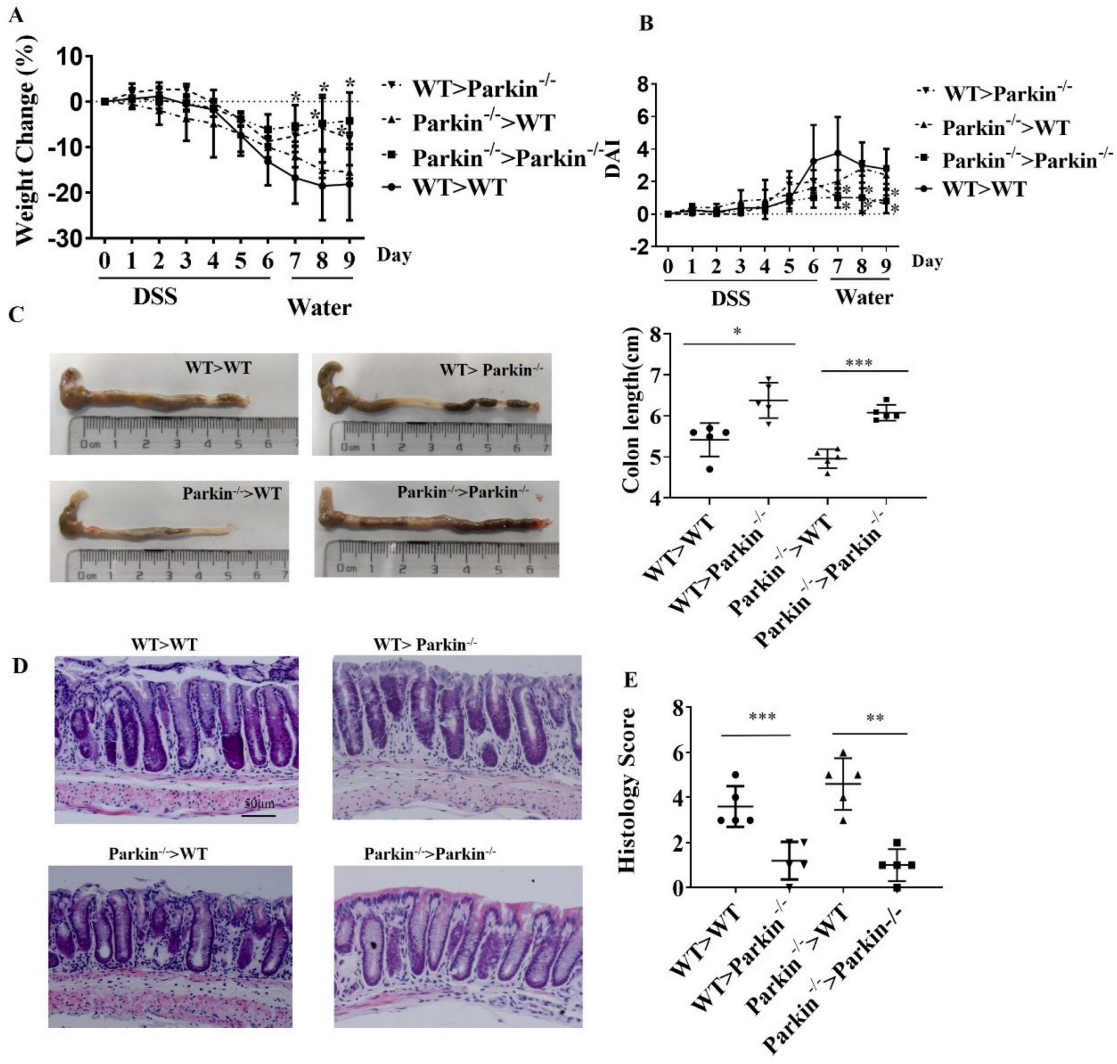
** Parkin-/- reduces colitis in a DSS-induced colitis model is independent of immune cells.** Lethally irradiated WT and Parkin-/- mice were reconstituted with WT and Parkin-/- bone marrow for 12 weeks, then were given 2.5 % DSS for six days, followed by normal drinking water for three days. Disease severity was measured by percentage weight loss(A), Disease activity index (DAI)(B), colon length (C), H&E-stained sections of the colon(D), scale bar=50μm, and histology score (E). Results are presented as means±SEM.*P < 0.05, **P < 0.01, *** *P* < 0.001 versus the corresponding WT>WT or Parkin^-/-^>Parkin^-/-^. n = 6-7 in each genotype.

Next, we examined whether alleviative colitis is related to the immune system of Parkin-/- mice. The donor bone marrow cells (BMCs) derived from Parkin deficiency mice were transplanted to recipes mice (WT or Parkin KO), or the donor BMCs derived from WT mice to recipes mice (WT or Pakin KO). These mice received seven days of DSS treatment in drinking water and two days containing water, and we recorded the body weight and clinical scores daily (Fig. 2A,2B). Colon length and Histological analysis showed no significant differences in these mice (Fig 2C-2E). These results indicate that Parkin-/- derived bone marrow does not affect the sensitivity of mice to DSS-induced colitis and that loss of Parkin reduces colitis independent of immune cells.

### Parkin interacts with VDR

**Figure 3:**
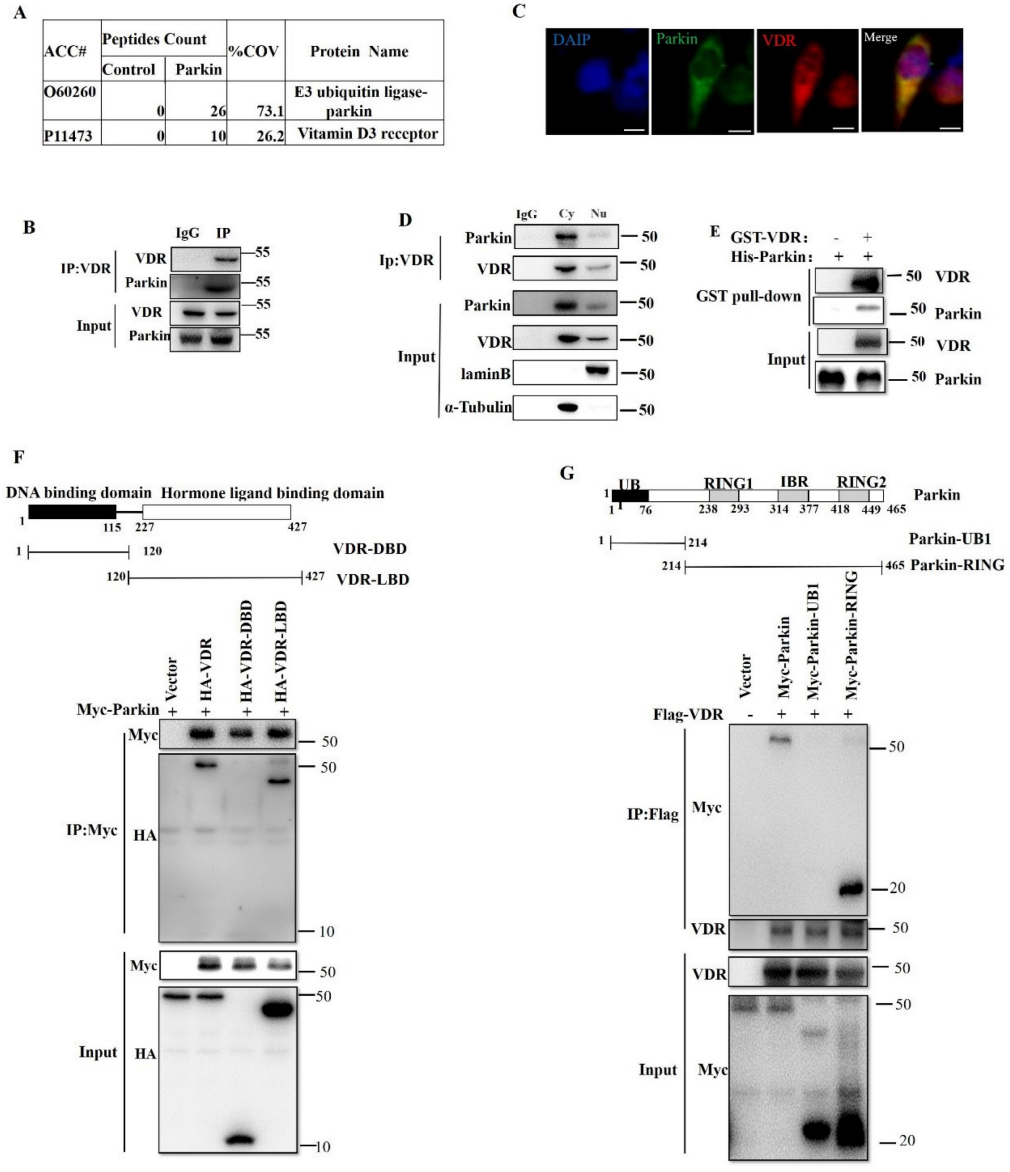
** Parkin interacts with VDR.** (A) Mass spectrometry analysis of Parkin and VDR peptides after immunoprecipitation with anti-Flag to pull down Parkin-associated proteins in Flag-Parkin over-expressed HCT116 cells. (B) Mice IECs lysates were immunoprecipitated (IP) and immunoblotted (IB) with the indicated antibodies. (C) Immunofluorescent staining for VDR and Parkin in HCT116. Scale bar: 20 μM. (D) Cytoplasm and nucleus of HCT116 were isolated, then immunoprecipitated (IP) and immunoblotted (IB) with the indicated antibodies. (E) Purified GST-VDR was incubated with purified His-Parkin for two h. His-Parkin bound to GST-VDR was pulled down by glutathione beads and subjected to immunoblot analysis with the indicated antibodies. (F) WT or mutant HA-VDR (DBD or LBD) and Myc-Parkin were expressed in HEK293T cells, immunoprecipitated, and analyzed by immunoblotting with the indicated antibodies. (G) WT or mutant Myc-Parkin (UB1 and Ring) and HA-VDR were transfected in HEK293T cells, immunoprecipitated, and analyzed by immunoblotting with the indicated antibodies. Data are representative of three independent experiments.

To find the possible mechanism of Parkin involved in IBD, we screened for potential Parkin-interacting proteins in the intestinal epithelial cell line HCT116. Parkin-associated protein complexes were immunoprecipitated and subjected to liquid chromatography-mass spectrometry for protein identification. One candidate protein of our interest is VDR (p11473), which is reported to inhibit colitis by protecting the mucosal epithelial barrier (Fig. 3A). Co-immunoprecipitation (co-IP) was used to confirm the interaction between endogenous VDR and Parkin in IECs (Fig. 3B). The overexpression of Flag-Vdr and HA-Parkin confirmed similar result in 293T cells Fig. S1A-B). By immunofluorescence assays, the colocalization of VDR and Parkin was observed in the cytoplasm (Fig. 3C), which was further confirmed by co-IP analysis of cytoplasmic and nuclear components (Fig. 3D). Moreover, GST pull-down assay indicated that Parkin directly interacted with VDR (Fig. 3E). To map the domain of Parkin or VDR required for their interaction, a series of co-IP assays were performed with truncated Parkin or VDR overexpressed in 293T cells (Fig. 3F and 3G). The results indicated that the C terminal of Parkin (including RING and IBR domain) and the ligand-binding domain (LBD) of VDR were sufficient for Parkin-VDR association. Collectively, these data fully demonstrate that Parkin interacts with VDR.

### Parkin downregulates the protein level of VDR

**Figure 4:**
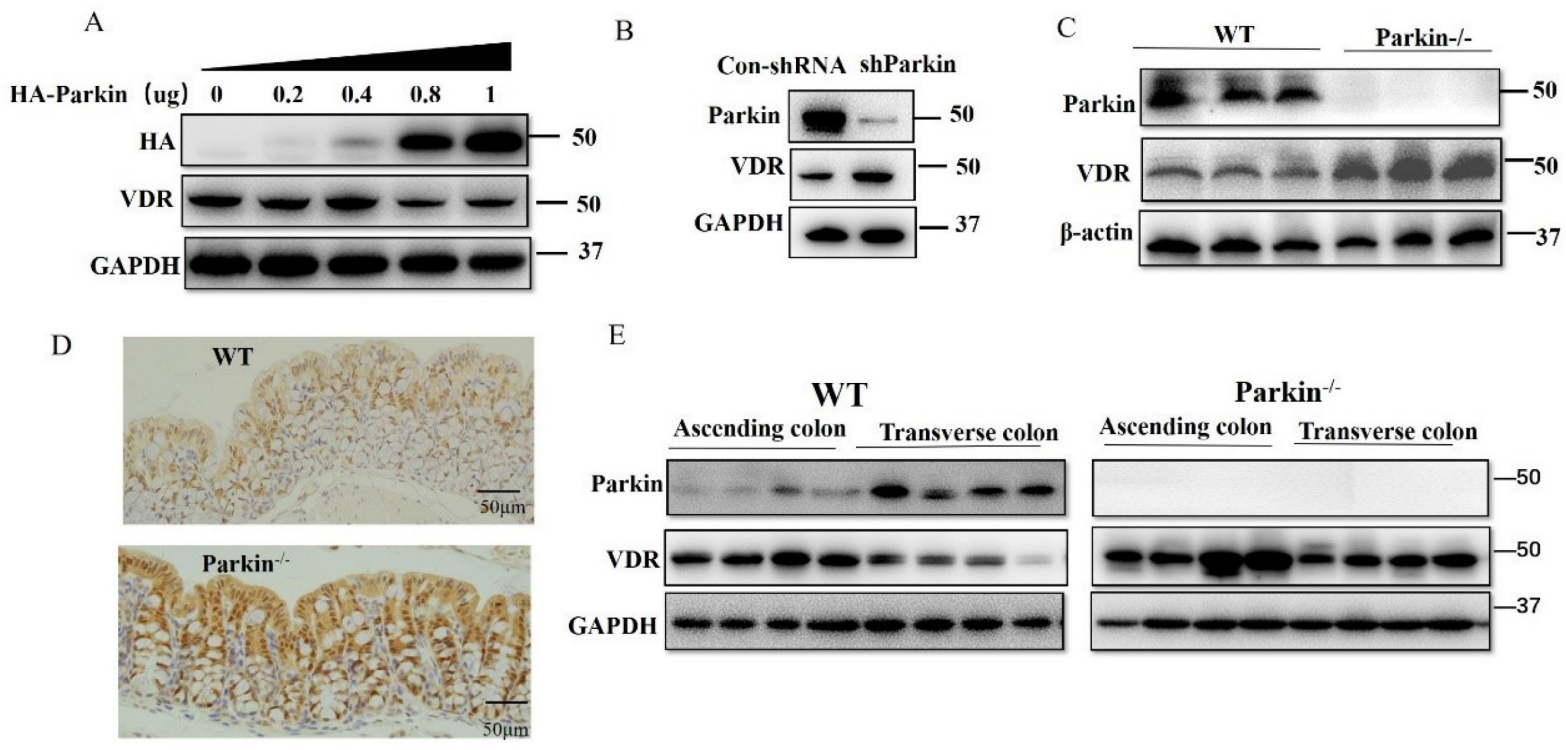
** Parkin downregulates VDR protein levels in intestinal epithelium cells.** (A) Varying amounts of HA-Parkin or empty control vectors were transfected into HCT116 cells and immunoblotted analysis with the indicated antibodies. (B) shRNA Parkin or control-shRNA was transfected into HCT116 cells and immunoblotted analysis with the indicated antibodies. (C) VDR expression in the colon of WT and Parkin-/- mice were analyzed by immunoblotting with the antibodies indicated. (D) Detecting VDR expression in the colon of WT and Parkin-/- mice by immunohistochemistry with anti-VDR; Scale bar=50μm. (E) Ascending colon and transverse colon were isolated from WT mice and analyzed by immunoblotting with the indicated antibodies.

For Parkin is an E3 ubiquitin ligase, we examined the effect of Parkin on VDR protein expression. In HCT116 cells, overexpression of Parkin decreased VDR protein level in a dose-dependent manner (Fig. 4A), and knockdown of Parkin increased VDR level (Fig. 4B). Similar result was also validated in CaCo2 cells (Fig. S2A) and Hela cells (Fig. S2B). Next, we detected VDR protein levels in IECs from Parkin-/- and WT mice. Data showed that VDR was highly expressed in colonic epithelial cells from Parkin-/- mice by western blotting and immunohistochemistry staining on colonic sections (Fig. 4C, 4D). Further examinations of tissues from ascending colon to the transverse colon in WT mice showed a negative correlation expression of Parkin and VDR* in vivo.* In contrast, in Parkin-/- mice, VDR protein level showed no apparent changes (Fig. 4E). In turn, Parkin protein level was similar in IECs from Vdr-/- and WT mice, suggesting that VDR does not regulate the protein expression of Parkin (Fig. S3. Together, these results indicate that Parkin negatively regulates VDR levels in the colonic epithelial cells.

### Parkin promotes VDR degradation via the autophagy-lysosome pathway

**Figure 5:**
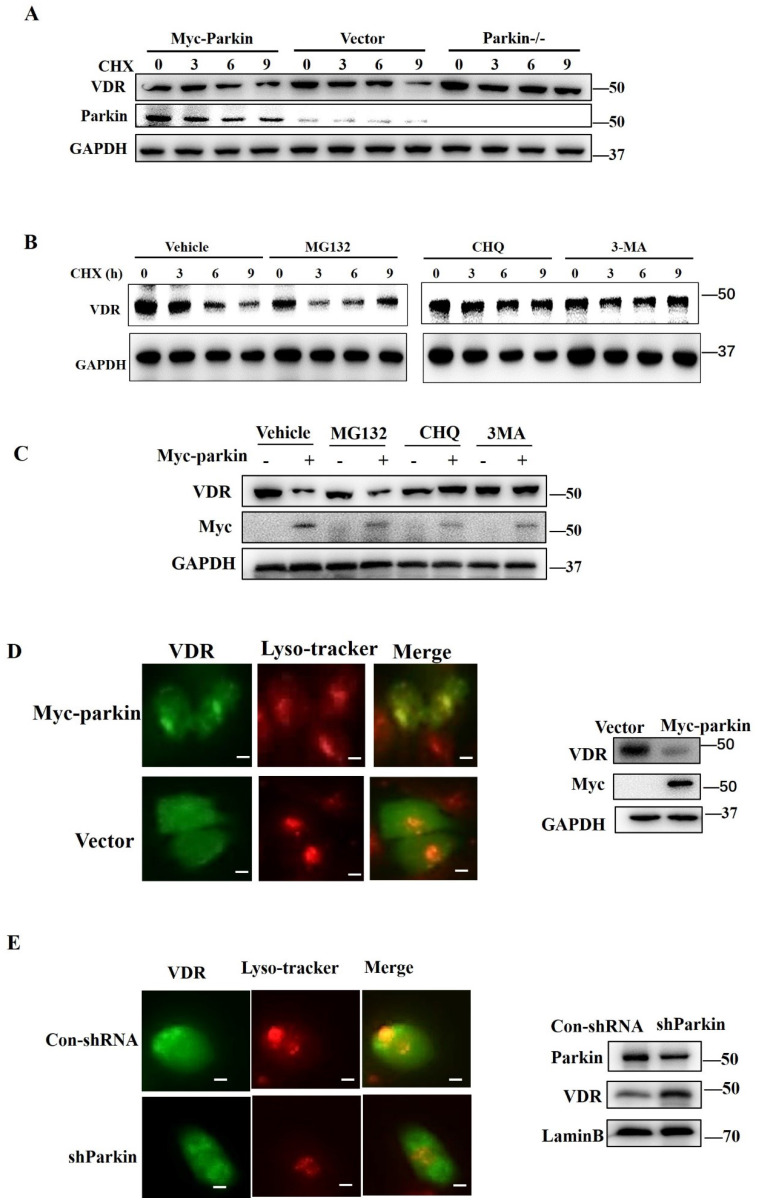
** Parkin decreases VDR protein expression via lysosome degradation.** (A) 50µg/ml CHX stimulated for indicated periods in HCT116 cells transfected with Myc-Parkin, shParkin, or control vector (Vect) into cells, then analyzed by immunoblotting with indicated antibodies. (B) MG132, CHQ, 3MA or control regent (Vehicle) pre-treated 30mins before CHX stimulated in HCT 116 cells, then CHX treatment for indicated period and detected endogenous VDR expression by Western blot. (C) MG132, CHQ, 3MA or control regent (Vehicle) treated HCT 116 cells transfected with Myc-Parkin or empty vector, then detected endogenous VDR expression by Western blot. (D) VDR and lysosomes were stained with anti-VDR (green) and Lyso-Tracker Red (C1046) in control shRNA or shParkin transfected HCT116 cells. Western blot assays detected Parkin and VDR expression; Scale bar=10 μM. (E) VDR and lysosomes were stained with anti-VDR (green) and Lyso-Tracker Red (C1046) in empty vector or Myc-Parkin transfected HCT116 cells. Western blot assays detected Parkin and VDR expression; Scale bar=10 μM.

To determine the function of Parkin in regulating VDR, we examined the transcriptional expression of VDR in the colon or HCT116 cells. The results showed that overexpression or knockdown of Parkin did not affect the level of Vdr mRNA Fig. S4A, S4B). CHX treatment led to rapid degradation of VDR when Parkin overexpression and Parkin knockdown attenuated the decrease of VDR (Fig. 5A). Considering that Parkin is an E3 ligase, we hypothesize that Parkin promotes VDR degradation in a ubiquitinated-dependent way. To prove this presupposition, we constructed Parkin mutants as S65A (21, T173A, and C431A 22, lacking the enzymatic activity of E3 ligase. However, these mutations could still downregulate the protein level of VDR, indicating that Parkin downregulates VDR, not through the ubiquitinated degradation pathway Fig. S5A). By co-IP assays, we confirmed that Parkin does not directly ubiquitinate VDR (Fig. S5B). Then we examined the protein level of VDR in the treatment of proteasome inhibitor MG132, lysosome inhibitor CHQ, or autophagy inhibitor 3MA, respectively (Fig. 5B). The decrease of VDR mediated by Parkin overexpression was effectively inhibited by CHQ and 3MA treatment (Fig. 5C), suggesting Parkin-induced VDR degradation is related to the autophagy-lysosome pathway. By immunofluorescence assay, we observed that Parkin overexpression promoted the recruitment of VDR into lysosome (Fig. 5D); on the contrary, the localization of VDR into lysosome was reduced by Parkin knockdown (Fig. 5E). These data suggest that Parkin induces VDR degradation via an autophagy-lysosome pathway.

### Parkin decreases VDR protein via P62

**Figure 6:**
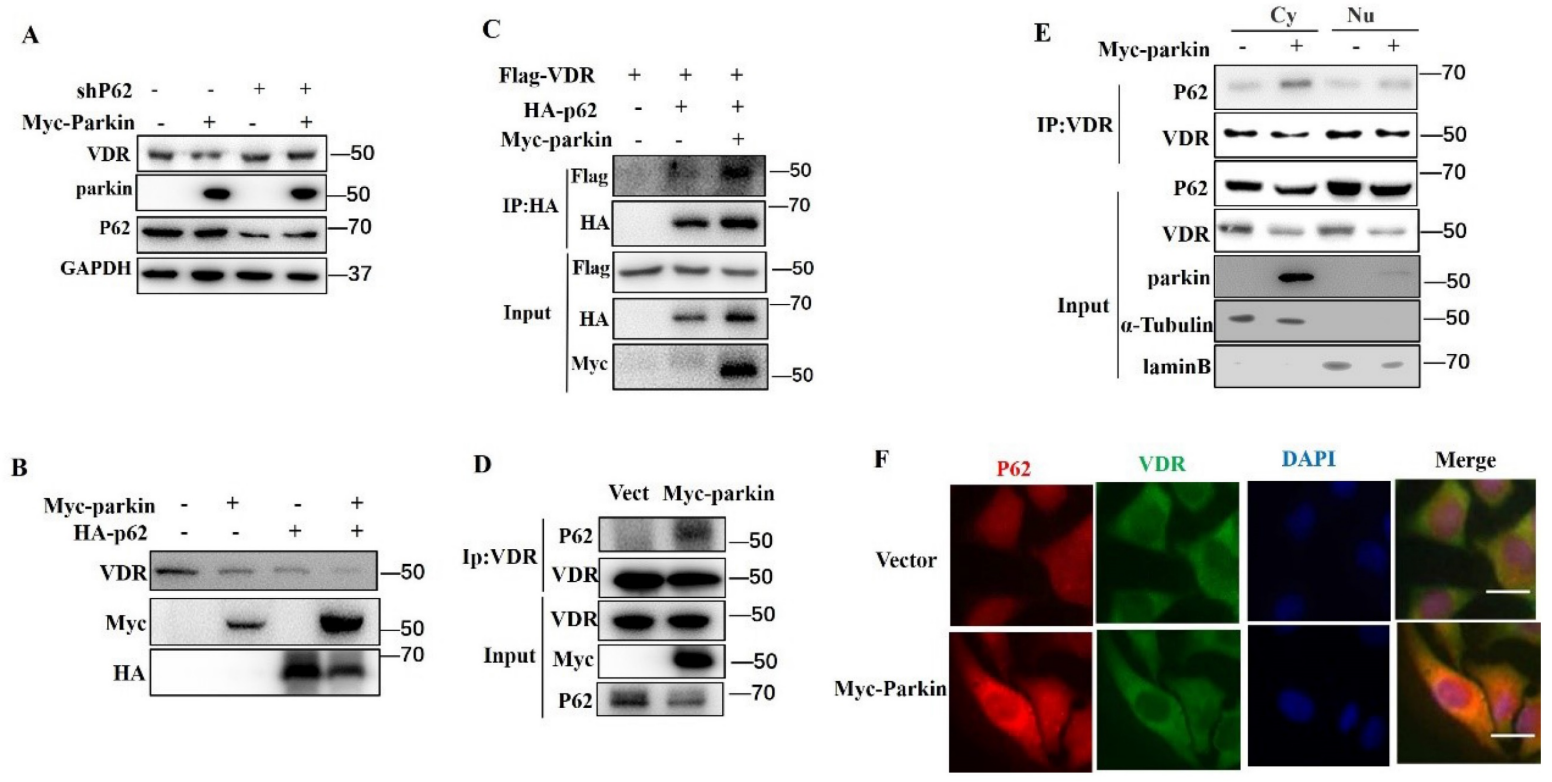
** Parkin decreases VDR protein via P62.** (A) shRNA P62 or Myc-Parkin was transfected into HCT116 cells as indicated and analyzed by immunoblotting with the indicated antibodies. (B) Myc-Parkin or HA-P62 was transfected into HCT116 cells as indicated and analyzed by immunoblotting with the indicated antibodies. (C) Flag-VDR, HA-P62, and Myc-Parkin were transfected into HCT116 cells as indicated, and HCT116 lysates were immunoprecipitated (IP) and immunoblotted (IB) with the indicated antibodies. (D) Myc-Parkin or control vector (Vect) was transfected into Hela cells, and cell lysates were immunoprecipitated (IP) and immunoblotted (IB) with the indicated antibodies. (E) Myc-Parkin or control vector (Vect) was transfected into HCT116 cells, isolated cytoplasm, nucleus, immunoprecipitated (IP), and immunoblotted (IB) with the indicated antibodies. Cy: cytoplasm; Nu: nuclear. (F) Myc-Parkin or control vector (Vect) was transfected into HCT116 cells and immunofluorescent stained VDR and P62 with anti-VDR and anti-P62; Scale bar=20 μM.

p62 is an adaptor that binds proteins or damaged organelles for their clearances utilizing autophagy-lysosome (12. In our experiments, the knockdown of p62 blocked the Parkin-mediated degradation of VDR (Fig. 6A). Consistently, overexpression of p62 enhanced the degradation of VDR induced by Parkin overexpression (Fig. 6B). To verify the involvement of p62 in the degradation of VDR mediated by Parkin, we performed co-IP experiments to validate the interaction between p62 and VDR. The results showed that Parkin overexpression effectively increased the VDR-p62 association, even endogenous VDR and p62 interaction (Fig. 6C, 6D). By co-IP analysis of cytoplasmic and nuclear extracts, the VDR-p62 association was observed in the cytoplasm (Fig. 6E). Similar result was also confirmed by immunofluorescence assays (Fig. 6F). Together, these results indicate that the Parkin-mediated VDR degradation is through the autophagy-lysosomal pathway by promoting the binding of VDR to p62.

### The role of Parkin in DSS-induced colitis via regulating VDR

**Figure 7:**
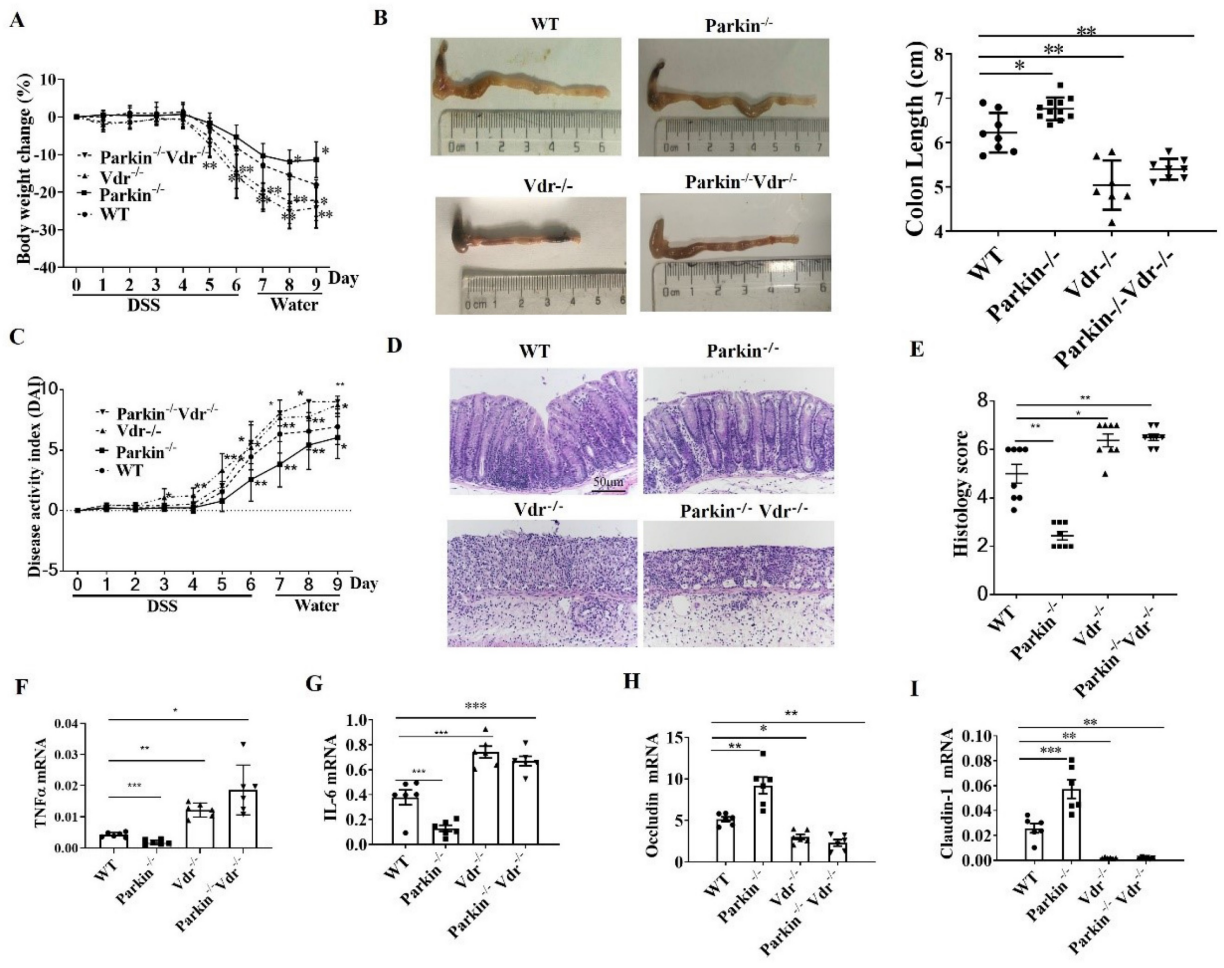
** Parkin inhibits DSS-induced colitis through VDR regulation.** WT, Vdr-/-, Parkin-/-, and Vdr-/-/Parkin-/- mice were given 2.5% (w/v) DSS for six days followed by normal drinking water for three days, and disease severity was measured according to a percentage weight loss(A), colon length(B), Disease activity index (DAI)(C), H&E-stained sections of the colon(D) and histology score (E), scale bar=50μm. Real-time RT-PCR quantitation of pro-inflammatory cytokines TNFɑ(F), IL-6(G), and tight junction protein Occludin(H), Claudin-1(I) in colonic mucosa from control and DSS-treated WT and Parkin-/-mice on day 9. Results are presented as means±SEM.*P < 0.05, **P < 0.01, *** *P* < 0.001 versus the corresponding WT. n = 6-7 in each genotype.

To investigate the role of Parkin-VDR pathway in the occurrence of colitis, we constructed Vdr-/-/Parkin-/- mice. DSS-induced colitis model was established in the following group: WT, Vdr-/-, Parkin-/-, and Vdr-/-/Parkin-/- mice. Compared with the WT group, Vdr-/- mice exhibited severe colitis with more significant body weight loss, shorter colons length, higher disease activity index (DAI), and histological scores (Fig. 7A-E). Meanwhile, Parkin-/- mice exhibited reduced inflammatory symptoms, but the symptoms of Vdr-/-Parkin-/-mice were the same as Vdr-/- mice, suggesting that VDR is the downstream target of Parkin (Fig. 7A-E). Consistent with these observations, the Vdr-/-Parkin-/- group increased the production of inflammatory cytokines and decreased tight junction proteins (Fig. 7F-I), completely reversing the effects caused by Parkin deficiency. Together, our data suggest the pathological role of Parkin in IBD via its regulation of VDR.

## Discussion

Preliminary studies indicate an anti-inflammatory effect of mitophagy on the gut micro-environment in IBD. The defect in Parkin-mediated mitophagy contributes to the accumulation of damaged mitochondria and drives inflammation 13. Here, we report a novel role of Parkin involved in the pathology of IBD by downregulation of VDR via p62-mediated autophagy-lysosome degradation. In experimental colitis models, Parkin-/- mice were highly resistant to colitis, but Vdr -/-Parkin -/- mice showed as severe inflammation as Vdr -/-mice. Our data demonstrate that the increased expression of VDR induced by Parkin-/- in epithelial cells protects the colonic mucosal barrier and attenuates colitis.

Many studies explore the Vdr gene as a known IBD risk gene 23,24. Vitamin D and VDR signaling play essential roles in the intestinal epithelium, where VDR signaling is linked to several crucial processes, such as cell proliferation, differentiation, immunomodulation, and maintenance of the gut microbiome 18,25,26. Since VDR is abundantly expressed in intestinal epithelial cells and plays a protective role in intestinal barrier integrity 25,27, the finding of Parkin-mediated degradation of VDR in IECs supports an additional role of Parkin in IBD, distinct from mitophagy. Notably, Parkin KO mice displayed multiple signs of inflammation and initial hyperplasia at 10 months old 14. We found that parkin KO mice aged 8-12 weeks were highly resistant to experimental colitis. This seemingly contradictory phenotype of Parkin deficiency in the IBD model is explicable. Compared with mice at 10 months old, VDR is highly expressed in young mice 28-30, so Parkin's regulation of VDR expression may take a leading role in the occurrence of enteritis prior to its role in mitophagy. These data indicate that the Parkin -VDR pathway in epithelial cells is the primary way for Parkin to be involved in IBD pathology.

We previously reported that TNF-α-induced downregulation of epithelial VDR was mediated by microRNA-346 31. However, more information on regulating epithelial VDR expression is largely unknown. This study reveals a novel mechanism for the regulation of VDR degradation. Parkin can function as an E3 ubiquitin ligase to ubiquitinate and degrade substrate proteins such as HIF-1ɑ 22, CDCrel-1 32, and synphilin-1 33. Here, we identify a novel pathway where Parkin does not depend on ubiquitination to induce VDR degradation via the autophagy-lysosome pathway. Angeles Duran has reported that p62 binds to VDR 34. Consistently, we found that the enhanced VDR-p62 interaction induced by Parkin was observed in the cytoplasm. The increased recruitment of p62 by the Parkin-VDR association helps to translocate the autophagy-lysosome. Therefore, Parkin induces VDR degradation through p62-mediated autophagy lysosomes, independent of ubiquitin-proteome degradation.

**Figure 8:**
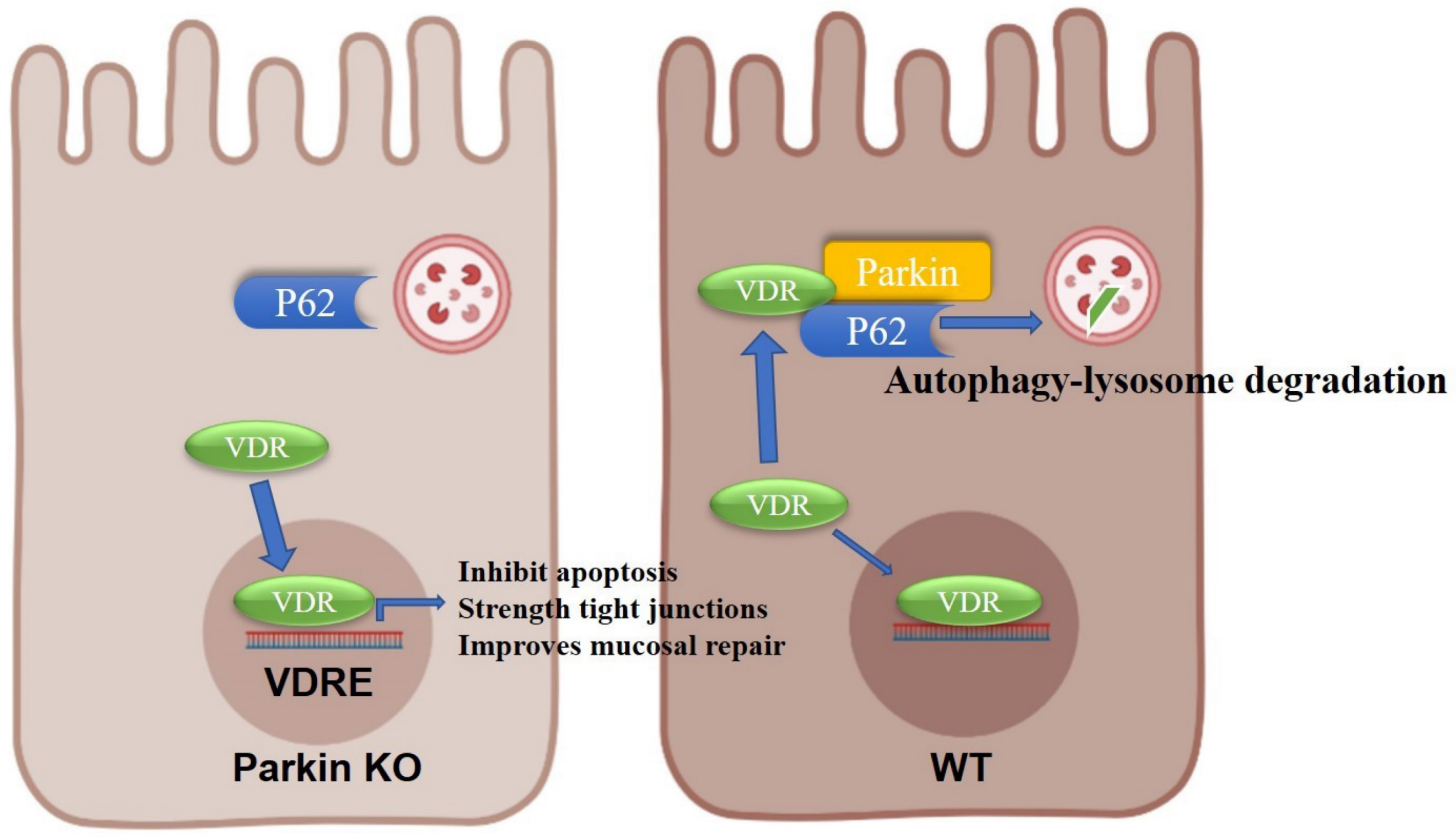
Model for the role of Parkin in regulating VDR degradation.

In conclusion, we provide evidence on the epithelial Parkin-VDR signaling in the pathogenesis of colitis (Fig. 8). For VDR level is related to many disease formations, monitoring Parkin expression might be an adjunct to the treatment of VDR-related diseases (such as fibrosis and cancer).

## Supplementary Material

Supplementary figures.

## Figures and Tables

**Table 1:** Primers

mGAPDH-1	5-GGGTGTGAACCACGAGAAATATG-3
mGAPDH -2	5-TGTGAGGGAGATGCTCAGTGTTG-3
mTNF-alpha -1	5-TCAGCCTCTTCTCATTCCTG-3
mTNF-alpha -2	5-CAGGCTTGTCACTCGAATTT-3
mIL-6-1	5-CCTCTCTGCAAGAGACTTCCA-3
mIL-6-2	5-AGAATTGCCATTGCACAACTCT-3
mOccludin-1	5-CCTCCAATGGCAAAGTGAAT-3
mOccludin-2	5-CTCCCCACCTGTCGTGTAGT-3
mClaudin1-1 mClaudin1-2	5- GATGTGGATGGCTCTCATTC-3 5-CGTGGTGTTGGGTAAGAGGT-3

## Abbreviations

IBD: inflammatory bowel diseases
VDR: Vitamin D receptors
CD: Crohn's disease
UC: ulcerative colitis
IEC: intestinal epithelial cell
PD: Parkinson's disease
Ubl: ubiquitin-like
OMM: mitochondrial outer membrane
BMCs: bone marrow cells
LBD: ligand-binding domain
WT: wild type
IP: Immunoprecipitation
IB: Immunoblot
DAI: Disease activity index
