# Supplementary material for: Parkin increases the risk of colitis by downregulation of VDR via autophagy-lysosome degradation
Source: Int J Biol Sci. 2023 Mar 5;19(5):1633–44. doi: 10.7150/ijbs.77153 (PMC10086757; doi:10.7150/ijbs.77153)

SUPPLEMENTAL INFORMATION

Figure S1. Parkin interacts with VDR in 293T.

Flag-VDR and HA-Parkin were expressed in HEK293T cells, and cell lysates were immunoprecipitated and analyzed by immunoblotting with the indicated antibodies (A, B).

Figure S2. Parkin expression downregulates VDR protein lever in dose.

HA-Parkin was transfected into CaCo2(A) and Hela(B) in dose, and cell lysates were analyzed by immunoblotting with the indicated antibodies.

Figure S3. VDR does not affect Parkin protein expression.

IECs lysates from Vdr-/- and WT mice were analyzed by immunoblotting with the indicated antibodies.

Figure S4. Parkin does not affect VDR mRNA expression.

(A) HC116 cells were transfected with varying amounts of HA-Parkin or empty vectors. Isolated mRNA from Cell lysates,qPCR analyzed the Vdr mRNA expression.

(B)Isolated mRNA from the WT and Parkin-/- mice colon,qPCR analyzed the Vdr mRNA expression.

FigureS5.Parkin downregulating VDR is independent of ubiquitin.

(A) Myc-Parkin WT, Myc-Parkin S65A, Myc-ParkinT173A, and Myc-Parkin C431A were transfected in HCT116 cells as indicated and analyzed by immunoblotting with the indicated antibodies.

(B) Flag-VDR, and Myc-Parkin were co-transfected with HA-ubiK48 or HA-ubiK48 in HCT116 cells as indicated and IP with HA, then analyzed by immunoblotting with the indicated antibodies.

Figure.S1

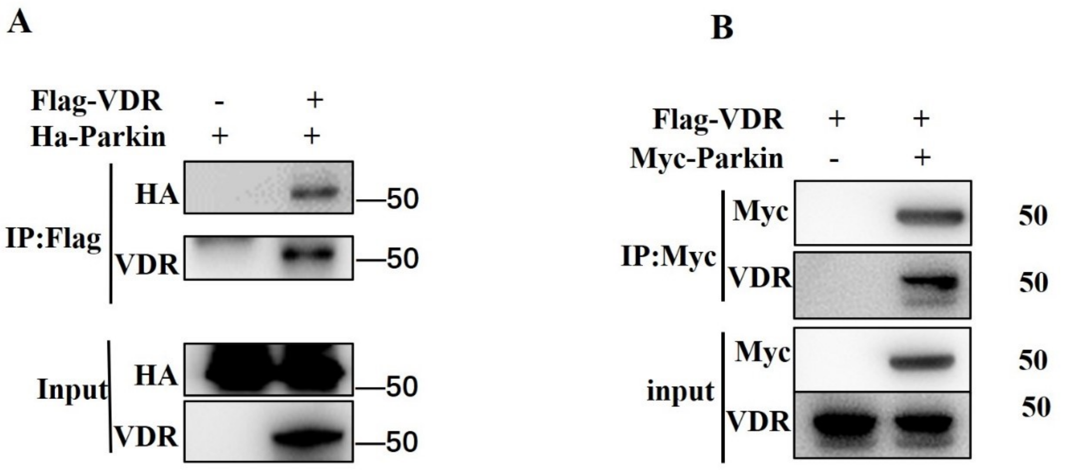

Figure.S2

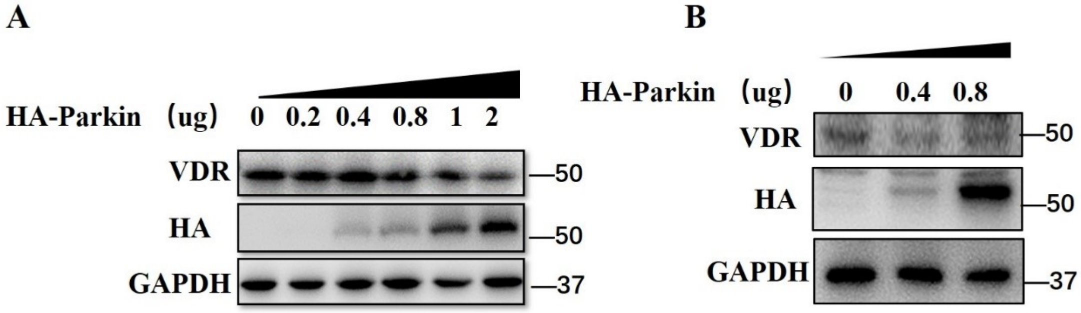

Figure.S3

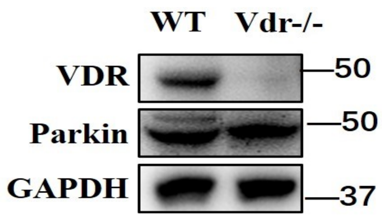

Figure.S4

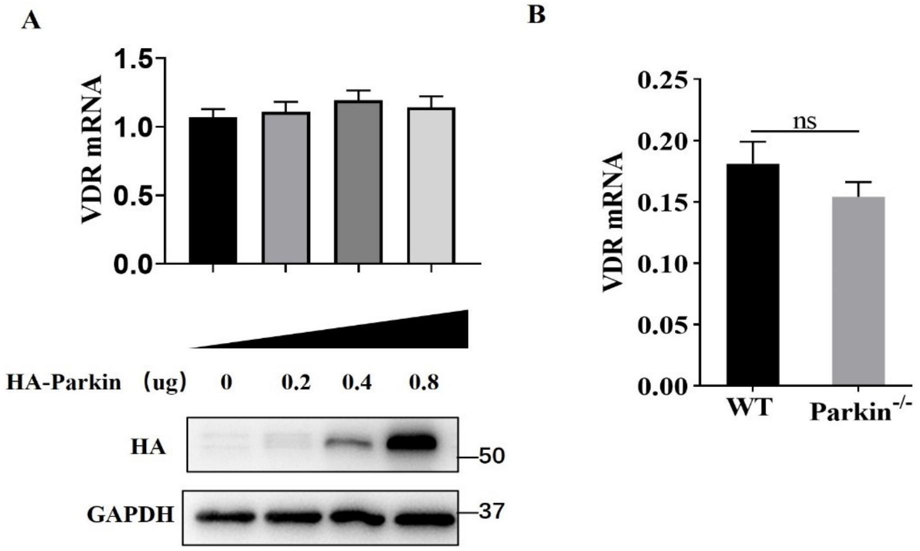

Figure.S5

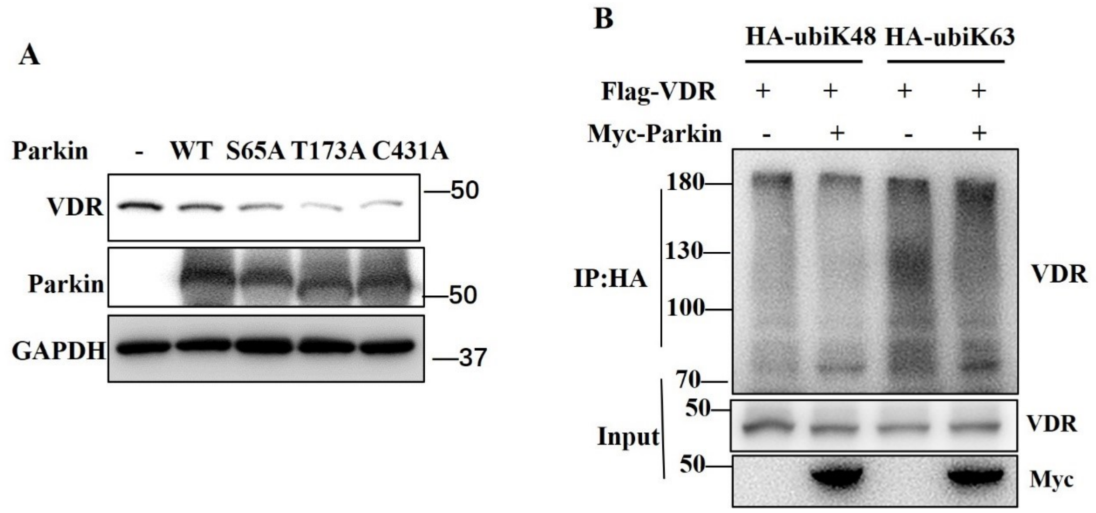

Supplement: Supplementary file 1 — Supplementary figures. [file ijbsv19p1633s1.pdf]
